# Influence of Ti-6Al-4V Scaffold Architecture on Early Cellular Responses Relevant to Bone Regeneration

**DOI:** 10.3390/jfb17090441

**Published:** 2026-09-01

**Authors:** Athanasios Armakolas, Amalia Kotsifaki, Martha Stathaki, Vassilis Paspaliaris, Eleni Tsakiri, Panagiotis Ntakos, Christos Kalligeros, Vasilios Spitas, Athanasios Foukas

**Affiliations:** 1 Physiology Laboratory, Medical School, National and Kapodistrian University of Athens, 11527 Athens, Greece; amkotsifaki@med.uoa.gr (A.K.); stathakimg@yahoo.gr (M.S.); vaspaspos@med.uoa.gr (V.P.); etsakiri@med.uoa.gr (E.T.); 2 Second Department of Internal Medicine at Hippokration General Hospital, National and Kapodistrian University of Athens, 11527 Athens, Greece; 3 Surgical Clinic, “Elena Venizelou” General Hospital, 11521 Athens, Greece; 4 Laboratory of Machine Design, National Technical University of Athens, Zografou, 15780 Athens, Greece; panosntak7@gmail.com (P.N.); ckalligeros@mail.ntua.gr (C.K.); vspitas@mail.ntua.gr (V.S.); 5 Third Department of Orthopaedic Surgery, “KAT” General Hospital of Athens, 14561 Kifissia, Greece; afoukas1@otenet.gr

**Keywords:** human mesenchymal stem cells (hMSC), 3D printing titanium scaffolds, porosity, surface area, bone regeneration

## Abstract

**Background**: Optimal porosity and surface area of additively manufactured Ti-6Al-4V scaffolds for early human mesenchymal stem cell (hMSC) retention remain unclear, despite their importance in reconstruction of critical-sized bone defects. This study evaluated scaffold architectures fulfilling biomechanical criteria for hMSC and osteocyte survival, proliferation, differentiation, cell cycle, and retention. **Methods**: Primary hMSCs from four healthy donors were characterized by flow cytometry, immunofluorescence, and Western blotting. Cells were seeded onto Ti-6Al-4V scaffolds with graded porosity (P50–P90, 50–90% porosity) and cultured for 120 h. Viability and retention were measured by trypan blue exclusion, apoptosis and cell cycle by flow cytometry, and osteogenic differentiation by collagen I, osteocalcin, and Akt phosphorylation analyses. Collagen-embedded hMSCs and osteoblasts were used to assess migration and phenotype maintenance. **Results**: The densest scaffold, P50, consistently retained significantly more viable hMSCs and differentiated osteoblasts than P60 and P70 (*p* < 0.005 in both cases, One-way ANOVA analysis, significance level a = 0.05 followed by Bonferroni correction). Among the scaffold architectures investigated, P50 demonstrated the most favorable early cellular retention under the present experimental conditions. No scaffold-induced apoptosis or proliferation changes were detected. Osteogenic differentiation and Akt phosphorylation increased during culture. In collagen-containing scaffolds, cells migrated from the matrix and preferentially coated titanium struts, with P50 supporting superior attachment. **Conclusions**: Among the scaffold architectures investigated, the low-porosity/high-surface-area P50 design provided the most favorable early microenvironment for hMSC and osteoblast retention without cytotoxic effects.

## 1. Introduction

Bone is a highly dynamic tissue that continuously undergoes remodeling in order to maintain its structural integrity and metabolic functions [1]. Beyond its structural and protective roles, bone functions as a mineral reservoir and is essential for hematopoiesis. These processes are maintained through the coordinated and balanced activity of osteoblasts, osteoclasts, and osteocytes [2]. Osteoblasts, which originate from mesenchymal stem cells (MSCs), are responsible for the synthesis and mineralization of the extracellular matrix [3]. Osteoclasts, derived from hematopoietic precursors, resorb bone tissue, while osteocytes regulate remodeling through mechanosensory and signaling functions [4]. The equilibrium between bone formation and resorption allows skeletal tissue to adapt to mechanical stimuli and repair microdamage throughout life [5].

Following loss of bone continuity in cases of trauma or following surgery for pathological condition such as infection neoplasia or congenital defects the regenerative capacity of bone may be severely challenged. Although bone possesses an intrinsic ability to heal, large or critical-sized defects frequently exceed this capacity and require clinical intervention either in the form of autologous bone graft or a reconstruction technique, such as Ilizarov, vascularized bone grafts or Masquelet, when bone gaps are larger than 5 to 6 cm [6]. Fracture healing is a complex biological process involving overlapping stages of inflammation, repair, and remodeling [7,8]. Immediately after injury, hematoma formation initiates a cascade of inflammatory events characterized by immune cell infiltration and cytokine release [9]. This early microenvironment is essential for recruiting progenitor cells, including MSCs, to the defect site [10]. During the reparative phase, these cells differentiate into chondrocytes and osteoblasts, forming a fibrocartilaginous callus that stabilizes the fracture [11]. Progressive mineralization then leads to the formation of a hard callus, which is gradually remodeled into mature lamellar bone [3]. While this process is highly efficient in small defects, extensive bone loss often necessitates supportive therapeutic strategies [3,10,11].

Autologous bone grafting remains the clinical gold standard for treating large osseous defects due to its osteogenic, osteoinductive, and osteoconductive properties [12,13]. However, it is associated with significant limitations, including donor site morbidity, limited tissue availability, prolonged operative time, and potential postoperative complications [11,12]. Furthermore, when bone gaps are larger than 5 to 6 cm, autologous bone grafts alone are not sufficient to promote bone union with contemporary surgical fixation techniques. Allografts and synthetic substitutes provide alternatives but may present reduced biological activity and risk of immune response and are equally ineffective in promoting bone union when bone defects are large [14,15,16]. These limitations have driven the development of tissue engineering approaches that integrate biomaterials, stem cells, and bioactive cues to enhance bone regeneration. Additionally, complex surgical techniques have been developed to optimize the biomechanical environment in complex cases with massive bone gaps [17].

Among the cellular components used in bone tissue engineering, MSCs play a pivotal role [18]. MSCs are multipotent stromal cells derived from various tissues, including bone marrow, adipose tissue, and perinatal sources [19]. They possess self-renewal capacity and the ability to differentiate into osteogenic, chondrogenic, and adipogenic lineages [20]. In the context of bone repair, their contribution extends beyond direct differentiation into osteoblasts [21]. MSCs exert substantial paracrine effects by secreting growth factors, cytokines, and extracellular vesicles that modulate inflammation, promote angiogenesis, and influence the behavior of resident bone cells [18]. These properties make them particularly attractive for regenerative applications.

Angiogenesis is a critical determinant of successful bone healing, as adequate vascularization ensures oxygen delivery, nutrient supply, and waste removal within the regenerating tissue [22]. MSCs contribute to this process by secreting pro-angiogenic factors, including vascular endothelial growth factor (VEGF), thereby facilitating the formation of new blood vessels within the defect site [20]. In addition, MSCs interact with osteoclast precursors through regulatory pathways, such as the RANKL/OPG system, contributing to balanced remodeling [23]. Their responsiveness to mechanical stimuli further enhances their osteogenic potential, as mechanical loading influences cytoskeletal organization and activates signaling pathways involved in bone formation [24].

The success of MSC-based therapies depends significantly on the microenvironment in which the cells are delivered [25]. In large bone defects, a suitable scaffold is required to provide structural support and guide tissue formation [26]. Titanium has long been used in orthopedic and dental applications due to its excellent mechanical properties, corrosion resistance, and biocompatibility [27]. Nevertheless, in cases where conventional solid titanium implants (plates and nails) are being used to treat large osseous defects final outcomes are not usually successful. Major problems include: mechanical failure of implants, fracture non-union, and infection [24,25,26]. For this reason, attention has shifted toward porous titanium structures that better mimic the architecture of cancellous bone [28].

Porous, additively manufactured titanium (Ti-6Al-4V) scaffolds have already moved beyond benchtop validation, with clinical reports and case series in large and massive segmental bone defects showing successful reconstruction and union when used as load-sharing graft containers [29]. Within titanium architected scaffolds, trabecular-mimetic Voronoi topologies have repeatedly been associated with strong osteogenic responses, with comparative in vitro/in vivo studies reporting equal or superior cell proliferation/osteogenic differentiation and bone formation versus common periodic or TPMS structures [30,31]. Accordingly, a logical next step is to move from uniform lattice designs to patient-specific, spatially graded architectures, where porosity (and thus local stiffness and pore-network space) is controlled topologically so that the resulting local strain/fluid-flow stimulus falls within mechanobiological windows that favor osteogenesis [32,33]. In parallel, this individualization must be mechanically constrained: graded porosity can be used to redistribute load and mitigate stress concentrations in critical regions [34,35], while satisfying strength/fatigue safety requirements and reducing failure risk in load-bearing use cases. Accordingly, there is a clear need to determine which porosity level yields the most favorable biological microenvironment, supporting MSC proliferation and osteogenic differentiation, while also maintaining robust osteoblast adhesion, so that porosity can be selected (and ultimately spatially graded) on a biology-informed basis rather than by purely geometric or mechanical criteria.

Recent advances in additively manufactured bone scaffolds have highlighted the importance of integrating scaffold architecture, material composition, and structural optimization to achieve an appropriate balance between mechanical performance and biological functionality [36]. In particular, current studies have explored the use of bioactive ceramic scaffolds with controlled three-dimensional architectures to enhance biomineralization and cellular responses, as well as gyroid-based interpenetrating lattice structures that enable greater tailoring of the physical and mechanical properties of implantable constructs [37]. Collectively, these developments demonstrate that scaffold topology and internal architecture are critical design parameters for the development of mechanically reliable and biologically supportive bone implants [36,37].

The interaction between MSCs and the scaffold surface is a critical determinant of regenerative success [34]. Surface topography and architectural features influence cell attachment, morphology, and cytoskeletal organization, thereby regulating intracellular signaling pathways associated with osteogenic differentiation [38]. In addition, scaffold geometry modulates local mechanical cues, further directing stem cell fate through mechanotransduction mechanisms [39,40]. Nevertheless, how structural porosity influences early MSC viability and functional stability remains unclear.

The integration of MSCs with structurally tailored 3D-printed titanium scaffolds has emerged as a promising strategy for addressing large and massive bone defects. However, achieving meaningful clinical benefit requires a scaffold design that not only fulfills anatomical and mechanical requirements but also actively supports stem cell survival, maintenance, and early functional activity. The present study therefore aims to compare a series of LPBF-manufactured Ti-6Al-4V scaffold architectures differing in porosity and available titanium surface area in order to identify the configuration that provides the most favorable early microenvironment for viable and functionally active hMSC retention prior to osteogenic commitment. Rather than identifying a universally optimal scaffold architecture for bone regeneration, the present study was designed to compare biologically relevant scaffold architectures under identical experimental conditions to determine which configuration best supports early hMSC retention and osteogenic cell stabilization.

By systematically assessing cellular attachment, distribution, viability, and early functional responses across scaffolds with distinct structural characteristics, this investigation seeks to define the configuration that provides the most favorable microenvironment for stem cell support. Defining this structural profile may clarify how scaffold architecture governs early MSC behavior and could guide future scaffold design strategies, ultimately contributing to improved healing in patients with critical-sized osseous defects.

Rather than identifying a universally optimal scaffold architecture for bone regeneration, the present study was designed to compare a series of biologically relevant LPBF-manufactured Ti-6Al-4V scaffold architectures under identical experimental conditions in order to determine which configuration provides the most favorable early microenvironment for hMSC retention and osteogenic cell stabilization.

## 2. Materials and Methods

### 2.1. Patients

Human bone marrow was obtained from the scraping material of four healthy donors aged 20–60 years with femoral fractures, or patients who had knee replacement surgery. The graft was collected at KAT Hospital, and the surplus graft (bone and bone marrow) was donated to the Physiology Laboratory. The material was used after the patients completed a written consent form. This study has been approved by the institution’s ethics committee, and all experimental procedures comply with the Declaration of Helsinki and have been explained to patients before surgery. Patients did sign a separate specific consent form before surgery to allow the use of their bone specimens for research purposes.

### 2.2. Isolation of Human Mesenchymal Stem Cells from Bone Marrow

The marrow was placed in a 100 mm cell culture dish with medium and disrupted using scalpels and scissors. The resulting pulp was filtered through a 70 mm mesh, and the resulting cells were cultured and allowed to grow for 7 days in modified Dulbecco’s Eagle’s medium (DMEM) and 20% fetal bovine serum (FBS). The culture medium was changed every 6 h for the first 4 days and then every other day. The cells were incubated in a standard tissue culture incubator (37 °C and 5% CO_2_). Before isolating HMSCs from the other bone marrow cell populations, we took advantage of the fact that HMSCs adhere only weakly to the plastic cell culture dish. Isolation was performed using 0.15% trypsin for 1 min at room temperature. The trypsin was then inactivated using growth medium; the detached cells were collected and cultured in 75 cm^2^ flasks until they reached a density of 25 × 10^5^ per 75 cm^2^ flask.

### 2.3. HMSC Characterization

The minimum criteria recognized by the International Society for Cell & Gene Therapy (ISCT) for the identification of human MSCs (hMSCs) are the expression of the markers CD73, CD90, and CD105 markers, and the absence of CD11b, CD14, CD19, CD34, CD45, CD79a, and HLA-DR markers. All these markers were examined by flow cytometry, flow cytometry analysis, and certain PCR markers and immunofluorescence.

### 2.4. Differentiation of HMSCs into Osteoblasts

For the differentiation of HMSCs into osteoblasts, StemPro Osteocyte Differentiation Basal Medium was used with the appropriate supplement for bone differentiation (GIBCO BRL). In human mesenchymal cells at a concentration of 80%, their nutrient medium was removed and replaced with the differentiation medium under the same incubation conditions. The medium was changed every three days. The complete differentiation of HMSCs into osteoblasts took 32 days. The stages of early and late (final) differentiation were monitored by immunofluorescence, detecting type I collagen FITC (early-stage differentiation) and osteocalcin Texas red (late-stage differentiation).

### 2.5. Scaffold Fabrication/Manufacturing

The five scaffold variants used in this study were generated as Voronoi lattice CAD/STL models differing in designed pore volume and available titanium surface area (P50, P60, P70, P80 and P90) and were manufactured from Ti-6Al-4V powder using laser powder bed fusion/selective laser melting (LPBF/SLM) on a Nikon SLM 280 2.0 system. Before printing, the STL files were checked and repaired, oriented within the build envelope, support structures were generated for mechanical stability and heat dissipation, and the models were sliced using Materialise Magics and the SLM Build Processor. For Ti-6Al-4V lattice production, the build was performed under an inert argon atmosphere using thin layers (20–60 um) and process settings selected within the supplier-reported operating window for fine lattice features: laser power 150–400 W, scan speed 600–1200 mm/s and volumetric energy density 50–120 J/mm^3^. The process window was selected for thin lattice struts in approximately 0.25–0.5 mm range, with local scanning strategies adjusted in lattice regions to limit overmelting, preserve strut geometry and avoid occlusion of the designed pores. The natural laser-generated surface topography was retained rather than mechanically polished. Following fabrication, the titanium scaffolds were sterilized by autoclaving prior to cell seeding. No chemical surface treatment was performed, thereby preserving the intrinsic laser-generated surface topography of the LPBF-fabricated scaffolds. Dimensional conformity of the printed scaffolds was assessed by optical laser scanning (ZEISS T-SCAN hawk 2) and nominal-to-actual comparison in ZEISS INSPECT against the source CAD models, with acceptance based on maintaining geometric deviations within +/−0.05 to ± 0.10 mm (Table 1 describes the structural details of each scaffold).

### 2.6. Flow Cytometry

The ISCT has proposed a minimum set of three standard criteria to be used as a uniform definition of pluripotent MSCs: (1) adhesion to plastic, (2) expression of specific surface antigens, and (3) multipotent differentiation potential. The phenotype of multipotent MSCs is defined as, at a minimum, the simultaneous expression on the cell surface of the antigens CD105, CD73, and CD90 antigens (≥95% positive) and the absence of hematopoietic lineage markers CD45, CD34, CD14 or CD11b, CD79a or CD19, and HLA-DR (≤2% positive). The ISCT has emphasized that the optimal flow cytometry assay uses multicolor analysis to show that individual cells co-express unique MSC markers and do not express hematopoietic antigens. Based on these data, we used a multicolor flow cytometry assay for hMSC analysis (Stemflow™ Human MSC Analysis Kit, BD Biosciences, San Jose, CA, USA) containing pre-conjugated and pre-titrated cocktails of positive expression markers defined by ISCT (CD105 PerCP-Cy™5.5/CD73 APC/CD90 FITC) and negative expression markers (CD45/CD34/CD11b/CD19/HLA-DR PE).

### 2.7. Apoptosis Detection

Cells were incubated for two days prior to Annexin staining. We used the FITC Annexin V Apoptosis Detection Kit I (BD Biosciences, Franklin Lakes, NJ, USA) before studying the effect of titanium scaffolds on the apoptosis of human mesenchymal stem cells. All samples were prepared according to the manufacturer’s instructions. Briefly, trypsin-attached cells and detached cells in culture medium were collected, combined, and washed with cold PBS. The pellets were resuspended in binding buffer, and 100 μL of each sample was used for staining with Annexin V-FITC and propidium iodide (PI). Staining was performed according to the protocol with 5 μL Annexin V-FITC and 5 μL PI for 15 min in the dark, and finally 400 μL of binding buffer (1×) was added to each tube. The samples were then analyzed using the BD FACSymphony A3 cell analyzer (BD Biosciences, Franklin Lakes, NJ, USA). Cells cultured on standard tissue culture plastic under identical culture conditions served as the control group, whereas the experimental group consisted of hMSCs cultured on the titanium scaffold architecture (P50) for the same incubation period. Following the identification of P50 as the scaffold demonstrating the highest cell retention, an apoptosis analysis was performed exclusively on this scaffold to determine whether the biologically most favorable architecture induced any cytotoxic effects compared with conventional monolayer culture. The cells were checked for debris and double cells. The populations of viable, early apoptotic, late apoptotic, and necrotic cells were quantified relative to single cells.

### 2.8. Cell Cycle Analysis

Cell cycle distribution was evaluated for HMSCs cultured in cell culture flasks and HMSCs grown on titanium scaffolds. Cells were inoculated two days prior to cell cycle analysis. After collection, the cells were washed twice with PBS and fixed by adding 75% cold ethanol dropwise while gently agitating to prevent clumping. The samples were stored at 4 °C for at least 1 h, washed twice to remove residual ethanol, then stained with PI/RNase staining buffer (BD Pharmingen; BD Biosciences, Franklin Lakes, NJ, USA) for 15 min at room temperature in the dark, and finally analyzed using the BD FACSymphony A3 Cell Analyzer (BD Biosciences, Franklin Lakes, NJ, USA). Cell-cycle distribution of hMSCs cultured on conventional tissue culture plastic was used as the control and compared with cells cultured on the P50 scaffold under identical experimental conditions.

### 2.9. Immunofluorescence Staining

Cultured cells on chamber slides were stained using the immunofluorescence method. The cells were washed in PBS and fixed with 80% ice-cold methanol for 10 min at room temperature. They were permeabilized with PBS plus 0.5% Triton X-100 (Sigma Aldrich, Burlington, MA, USA) for 10 min. They were then incubated with primary antibodies at room temperature: CD105 PerCP-Cy™5.5/CD73 APC/CD90 FITC, and CD44 PE to determine and certify mesenchymal cells, as well as collagen I FITC to determine the early differentiation of mesenchymal cells into osteoblasts, and osteocalcin Texas red to determine the complete conversion of stem cells into osteoblasts. After three washes, the samples were stained with DAPI (1 μg/mL) for observation under a two-photon confocal microscope (ZEISS LSM 900 Confocal Microscope, Carl Zeiss Microscopy GmbH, Jena, Germany).

Parallel cultures maintained on standard tissue culture plastic were used as controls for all immunofluorescence analyses to confirm marker expression independently of scaffold architecture. In order to determine the optimum scaffold structure that will allow the growth and differentiation of human hMSC into bone, hMSCs then placed on different titanium alloy scaffolds in regard to their porosity and their internal titanium area, at a density of 1 × 10^5^ cells/scaffold. A total of 1 × 10^5^ hMSCs were initially seeded onto each scaffold. Each sterilized scaffold was individually placed into a well of an 8-well chamber slide, where the scaffold closely fitted the dimensions of the well. A suspension containing 1 × 10^5^ hMSCs in 100 μL of complete culture medium was carefully pipetted directly onto the upper surface of each scaffold. The constructs were incubated for 3 h at 37 °C in a humidified atmosphere containing 5% CO_2_ to allow initial cell attachment to the titanium surface. Following the attachment period, each scaffold was carefully transferred to an individual well of a 24-well culture plate, and complete culture medium was gently added for subsequent culture under standard conditions.

The cell numbers represent the number of viable cells recovered and retained on each scaffold at the indicated time points (0, 24, 48, 72, and 120 h), rather than the initial seeding density. These scaffolds with the cells were incubated at 37 °C and 5% CO_2_ in a standard cell culture chamber. The viability, the expansion rate and the stability of these hMSC on each titanium scaffold was then examined in 4 and 8 days. Each scaffold was then washed, and the remaining cells on the dishes were collected after treatment with trypsin and counted using trypan blue analysis. The initial seeding density was identical for all scaffold groups (1 × 10^5^ cells/scaffold), allowing direct comparison of viable cell retention between scaffold architectures.

### 2.10. Western Blot Analysis and Densitometric Analysis

Akt activation: We analyzed the activation (phosphorylation) of Akt-kinase (Protein kinase B) in HMSCs along their differentiation process towards osteoblasts, by western analysis as described elsewhere [41]. Briefly, HMSC and differentiated hMSC cells towards bone at different stages, were analyzed (days 0, 10 and 20). Cell extracts were obtained by cell lysis in RIPA buffer containing protease and phosphatase inhibitors (SIGMA). RIPA buffer (100 μL per well) was added in each well of a 24-well plate and the plate was placed on a shaker for 20 min at 4 °C. Equal amount of cell lysates (60 μg) were heated at 95 °C for 5 min, electrophoresed on SDS–PAGE under denaturing conditions using a 12% acrylamide gel and transferred onto nitrocellulose membrane (Bio-Rad Laboratories, Hercules, CA, USA). The membranes were incubated overnight at 4 °C with primary rabbit anti-human antibodies against phosphor-Akt (Ser 473) and total Akt and GAPDH (1:1000 dilution for all, Cell Signaling, Beverly, MA, USA). Detection was carried out by using an HRP goat anti-rabbit IgG (1:2000 dilution; Santa Cruz Biotechnology, Dallas, TX, USA) and by using the Supersignal West Pico Chemiluminescent substrate kit (Thermo Scientific, Waltham, MA, USA).

Western blot bands were subjected to semi-quantitative densitometric analysis using ImageJ software (version 1.54; National Institutes of Health, Bethesda, MD, USA). For each lane, the integrated density of the pAkt and tAkt bands was measured following background subtraction and normalized to the corresponding GAPDH band intensity from the same sample. The resulting pAkt/GAPDH and tAkt/GAPDH ratios were subsequently expressed relative to the undifferentiated hMSC control group (day 0), which was assigned a value of 1.00. Data are presented as relative fold expression (mean ± SD).

### 2.11. Preparation of Collagen-Containing Titanium Scaffolds

For the collagen-based experiments, Rat Tail Collagen I (ibidi GmbH, Gräfelfing, Germany) was prepared according to the manufacturer’s instructions. HMSCs or differentiated osteoblasts were suspended in the collagen solution prior to gel polymerization. An equal volume of the collagen–cell suspension was carefully introduced into each porous titanium scaffold to promote homogeneous infiltration throughout the scaffold architecture. The constructs were then incubated at 37 °C in a humidified atmosphere containing 5% CO_2_ for 1 h to allow complete collagen polymerization, after which complete culture medium was gently added. The collagen-containing scaffolds were subsequently maintained under standard culture conditions (37 °C, 5% CO_2_), and the culture medium was replaced every 2–3 days according to the experimental protocol.

### 2.12. Statistical Methods

The results obtained by the trypan blue analyses, Annexin PI and cell cycle analysis, were assessed by One-way ANOVA analysis, followed by Bonferroni correction (SPSS v. 11 statistical package); SPSS RRID:SCR_002865, [IBM, Armonk, NY, USA]. Statistical significance was set at a = 0.05, i.e., *p* values less than 0.05 (* *p* < 0.05) were considered statistically significant. Comparisons between groups were accordingly performed using Student *t* test and One-way ANOVA analysis, followed by Bonferroni correction. A * *p*-value < 0.05 was considered statistically significant. Statistical significance was defined as follows: *p* < 0.05 (*); *p* < 0.005 (**).

## 3. Results

### 3.1. Identification of Mesenchymal Cells (MSC)

**Figure 1:**
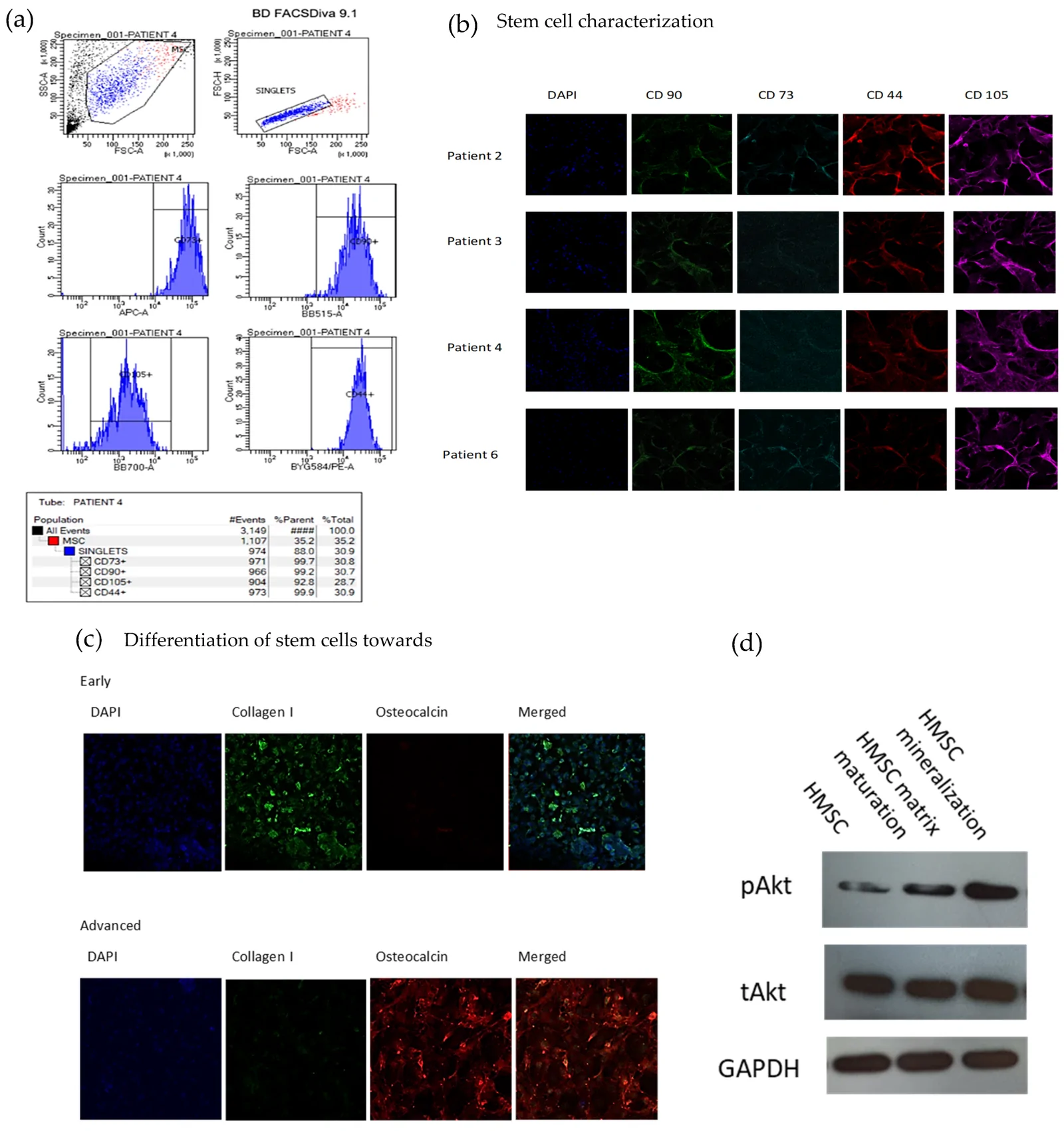
Characterization of HMSC: (**a**) Isolated HMSC were examined for their identity by flow cytometry where the cells were stained positively stained with CD73, CD90, CD105 and CD44. (**b**) Immunofluorescence: HMSC isolated from all four patients were also characterized by immunofluorescence for the markers CD90, CD73, CD44 and CD105. (**c**) In vitro differentiation of HMSC towards Bone was obtained by changing the growth media to osteogenic media. The differentiated cells were stained with two different markers Collagen I, which indicates early differentiation and Osteocalcin which indicates late differentiation towards bone (mineralization) at days 0, 10 and 20 respectively. (**d**) Western blot analysis of the phosphorylated Akt. The phosphorylated AKT indicates the active differentiation process. Suggesting that Akt is driving both early commitment and late-stage mineral deposition. Experiments were performed using four independent donors (*n* = 4), each measured in triplicate.

The results obtained by flow cytometry on isolated primary cells for their identification showed that in each case the isolated cells were of the mesenchymal type, as they met three standard criteria according to the ISCT that define the multipotency of MSCs: (1) adhesion to plastic, (2) expression of specific surface antigens, and (3) multipotent differentiation potential. The phenotype of multipotent MSCs is defined as, at a minimum, the simultaneous expression on the cell surface of the antigens CD44, CD105, CD73, and CD90 (≥90% positive) and the absence of hematopoietic lineage markers CD45, CD34, CD14 or CD11b, CD79a or CD19, and HLA-DR (≤2% positive) (Figure 1a). These results were also confirmed by immunofluorescence (Figure 1b).

### 3.2. Differentiation of HMSCs into Osteoblasts

To investigate the interaction between titanium scaffolds and osteoblasts, representing the final stage of mesenchymal stem cell differentiation during bone formation, HMSCs were differentiated into osteoblasts. Successful differentiation was monitored by immunofluorescence staining for type I collagen (FITC), as a marker of early osteogenic differentiation, and osteocalcin (Texas Red), as a marker of late-stage osteogenic differentiation (Figure 1c).

### 3.3. Akt Phosphorylation During Osteogenic Differentiation

One essential factor for osteogenesis is the activation of the PI3K/Akt pathway. Therefore, Akt phosphorylation is crucial for osteoblast differentiation, matrix mineralization, and ALP activity of HMSC, and its levels are increased as the differentiation proceeds and it is decreased when differentiation is completed [32]. In our experiment an increase in the pAkt is observed even at the mineralization stage of the differentiation indicating that differentiation is still an ongoing process (Figure 1d). Semi-quantitative densitometric analysis was performed by normalizing the background-corrected band intensity of pAkt and tAkt to the corresponding GAPDH signal within each lane. The resulting pAkt/GAPDH and tAkt/GAPDH ratios were subsequently expressed relative to the undifferentiated hMSC group, which was assigned a value of 1.00. This analysis demonstrated a progressive increase in relative pAkt expression during osteogenic differentiation (1.00 ± 0.10, 1.85 ± 0.18, and 3.10 ± 0.25 for hMSCs, matrix maturation, and mineralization, respectively), whereas the normalized tAkt expression remained essentially unchanged (1.00 ± 0.08, 0.98 ± 0.09, and 1.02 ± 0.10, respectively) (Table 2).

### 3.4. Plain Titanium Scaffolds

**Figure 2:**
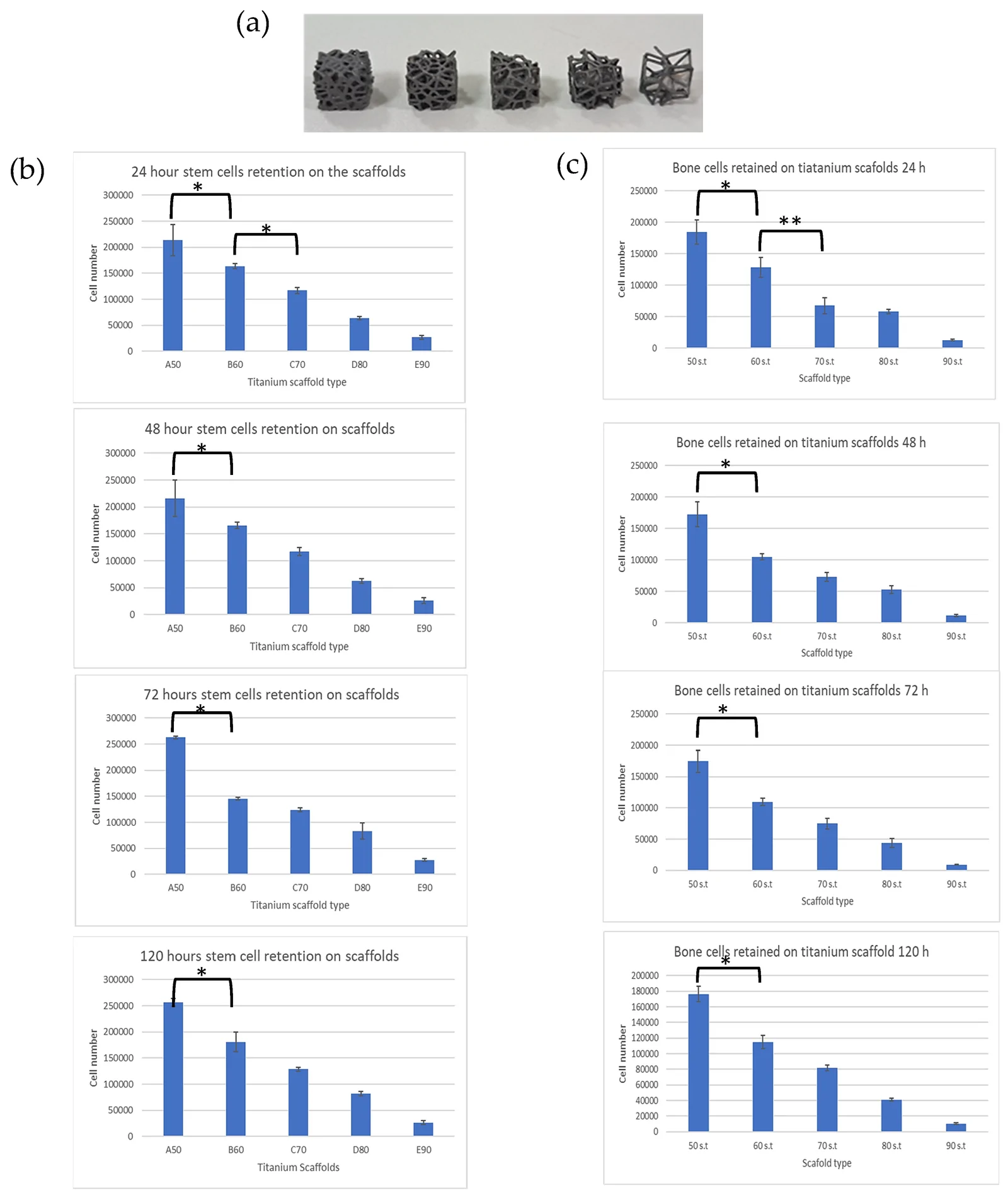
(**a**) Representation of the five different titanium scaffolds tested in this study. (**b**,**c**) Trypan blue analysis: Each of these scaffolds was co-cultured with HMSCs and Bone cells (Late stage differentiated HMSC towards bone) in order to determine the ability of each scaffold to retain the cells. In every case, P50, the denser scaffold appeared to present a significantly higher retention of both cell types. Experiments were performed with four independent donors (*n* = 4), with each measured in triplicate. * *p* < 0.05; ** *p* < 0.005.

Five different, mTi-6Al-4V Voronoi scaffolds, in respect to porous size and surface, were used in order to determine the optimal scaffold configuration in terms of cellular incorporation and differentiation towards osteoblasts and bone formation, (Figure 2a).

#### 3.4.1. Trypan Blue Analysis

Trypan blue analysis was performed to quantify the number of viable cells retained on each scaffold at 0, 24, 48, 72, and 120 h after the initial seeding of 1 × 10^5^ cells/scaffold. It was observed that the largest number of cells was retained by the densest titanium scaffold (P50). At 24 h, P50 retained approximately 27% more viable hMSCs than P60 and 75% more than P70, with statistically significant differences between P50 and P60 (* *p* < 0.05) and between P60 and P70 (** *p* < 0.005) at each individual time point, as determined by one-way ANOVA followed by Bonferroni post hoc correction. Similar differences were observed throughout the remaining time points. The experiments were performed separately for each patient with similar results. Data represents four independent donors (*n* = 4), each measured in triplicate (Figure 2b).

Trypan blue analysis was also performed on osteoblasts to determine the number of live bone-differentiated cells that can remain in the different structures of the titanium scaffolds. In this case, measurements were also performed for 120 h (0, 24, 48, 72, 120) for all titanium scaffolds and for all patients. It was observed that the most effective structure was again the densest scaffold, P50, showing significantly higher cell retention compared to P60 (* *p* < 0.05) and P60 compared to P70 (** *p* < 0.005) at each time point separately (One-way ANOVA analysis). Data represents four independent donors (*n* = 4), each measured in triplicate (Figure 2c).

To distinguish the effect of scaffold architecture on early cell retention from its effect on cell proliferation, the relative growth rate between 24 h and 120 h was also calculated (Table 3). Although P50 consistently retained the highest absolute number of viable cells throughout the experiment, comparison of the relative growth rates indicated that scaffold architecture primarily influenced early cellular retention rather than intrinsic proliferative capacity.

#### 3.4.2. Determination of the Toxicity of Plain Titanium Scaffolds

**Figure 3:**
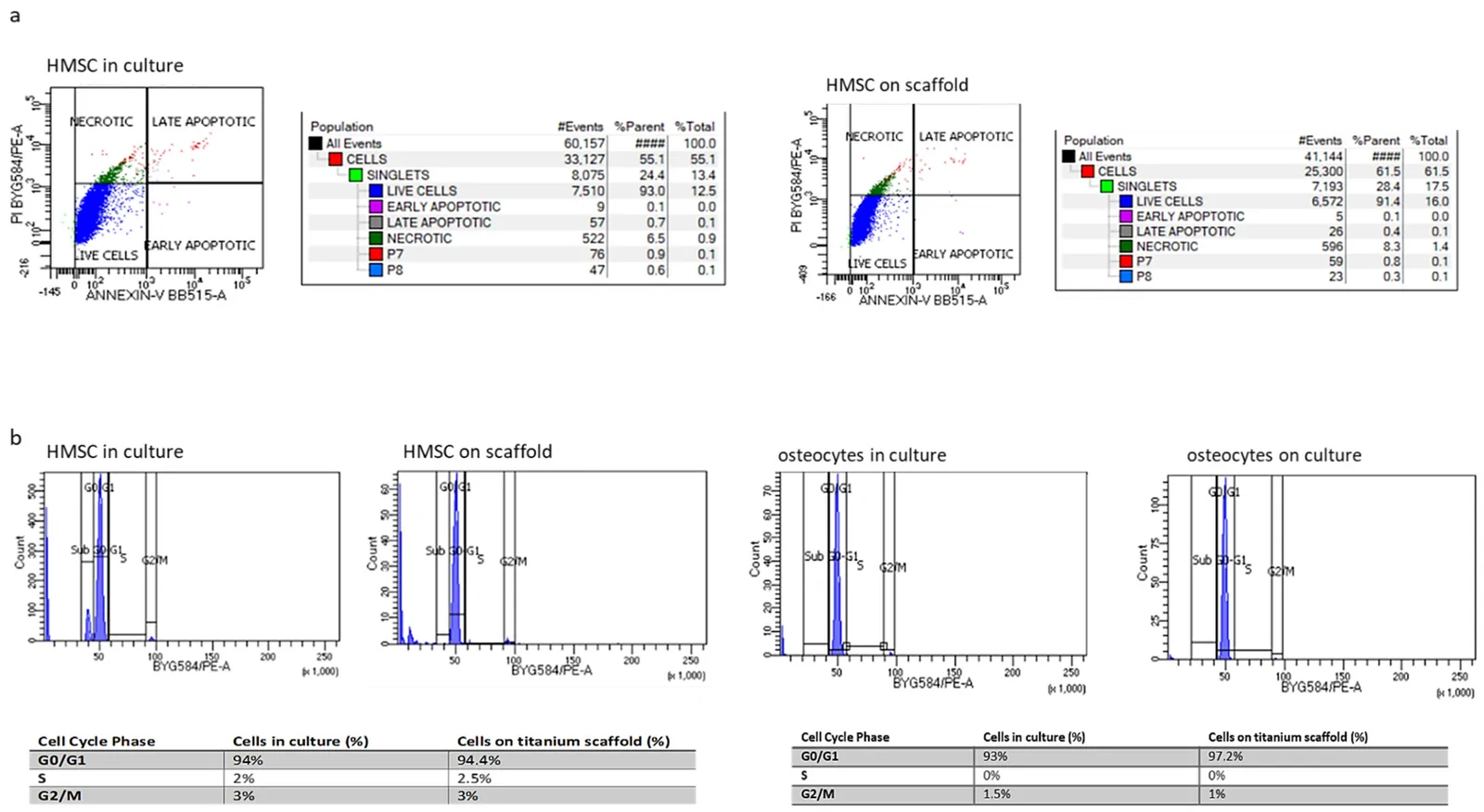
Determination of toxicity induced by the titanium scaffolds: hMSCs and bone cells were cultured after their differentiation on the scaffolds for four days. The results were compared with the cells in culture. (**a**) Annexin PI: No significant increase in apoptosis and necrosis was observed in any case. (**b**) Cell cycle analysis: The titanium scaffold did not seem to affect the cell cycle of either the HMSC or the bone cells in any case. Experiments were performed using four independent donors (*n* = 4), each measured in triplicate.

The analysis of live and apoptotic mesenchymal cells from the four patients was performed only for the P50 scaffold, which proved to be the most effective in retaining mesenchymal and osteoblasts, using the Annexin/Propidium Iodide technique. Following identification of P50 as the scaffold demonstrating the highest cellular retention, apoptosis analysis was performed exclusively on this architecture to determine whether the biologically most favorable scaffold induced any cytotoxic effects compared with conventional cell culture. The results suggest that there were no statistically significant differences between the cells in culture and the cells on the scaffolds, thus indicating that titanium scaffolds do not promote cell death in mesenchymal cells (Figure 3a).

#### 3.4.3. Determination of Induction or Suppression of the HMSC Cell Cycle by Plain Titanium Scaffolds

The purpose of the apoptosis and cell-cycle experiments was not to compare scaffold architectures but to determine whether the scaffold exhibiting the best biological performance (P50) exerted any cytotoxic or antiproliferative effects. Cell cycle analysis with propidium iodide showed no differences in the percentages of cells in each phase of the cell cycle between HMSCs derived from the four patients cultured without titanium scaffolds and HMSCs from the same patients cultured on P50 titanium scaffolds, suggesting that the P50 titanium scaffold does not affect HMSC proliferation. The results are described in Figure 3b.

#### 3.4.4. Titanium Scaffolds with Collagen

**Figure 4:**
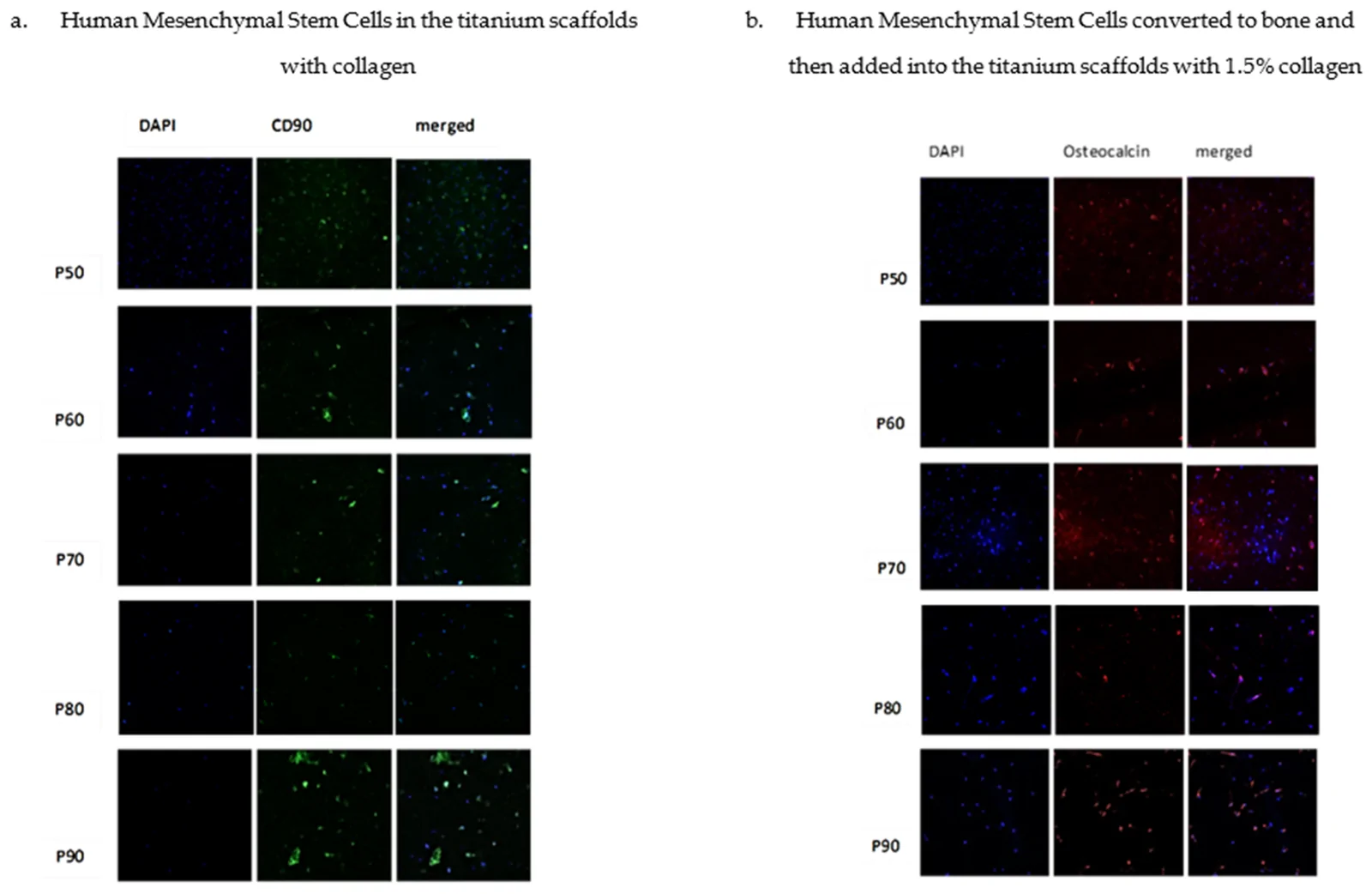
Introduction of hMSCs and differentiated osteoblasts into collagen-containing titanium scaffolds. (**a**) Representative immunofluorescence images of hMSCs cultured within 1.5% collagen-containing titanium scaffolds for 7 days, demonstrating cell viability, localization, and maintenance within the collagen matrix. Cell nuclei were stained with DAPI (blue), while CD90-positive hMSCs are shown in green. (**b**) Representative immunofluorescence images of differentiated osteoblasts cultured under the same conditions for 7 days, confirming preservation of cell viability and osteogenic phenotype within the scaffold. Cell nuclei were stained with DAPI (blue), while Osteocalcin expression is shown in red. Merged images show the combined fluorescence channels. These images are qualitative and illustrate cellular localization within the collagen-containing scaffolds rather than quantitative differences in cell retention between scaffold architectures. Experiments were performed using four independent donors (*n* = 4), each measured in triplicate.

Aiming to determine the actual effect of the different titanium scaffolds in the bone progression and prior to resemble the natural course of the bone repair we used the same titanium scaffolds but with 1.5% collagen. This collagen concentration would allow the stem cells to move freely, in and out of the scaffold allowing us to determine the friendliest structure for HMSC and osteoblasts. On these grounds two different experiments were obtained in the first case we introduced stem cells into the collagen, and we observed their migration capacities and viability and in another set of experiments we introduced fully differentiated hMSCs towards bone into the collagen of the titanium scaffolds and we determined if these cells retain their bone phenotype after culturing their number and viability. It was found, by immunofluorescence staining, that cells in both cases (hMSCs and bone cells remained in the collagen in the scaffold, and they were alive and functional since they produced the proteins stained (Figure 4a,b). Representative immunofluorescence analysis demonstrated osteocalcin expression in all scaffold architectures. Although the absolute number of retained cells differed among scaffolds, osteogenic marker expression was preserved across all groups, indicating that scaffold architecture did not impair the osteogenic differentiation capacity of the surviving cells.

**Figure 5:**
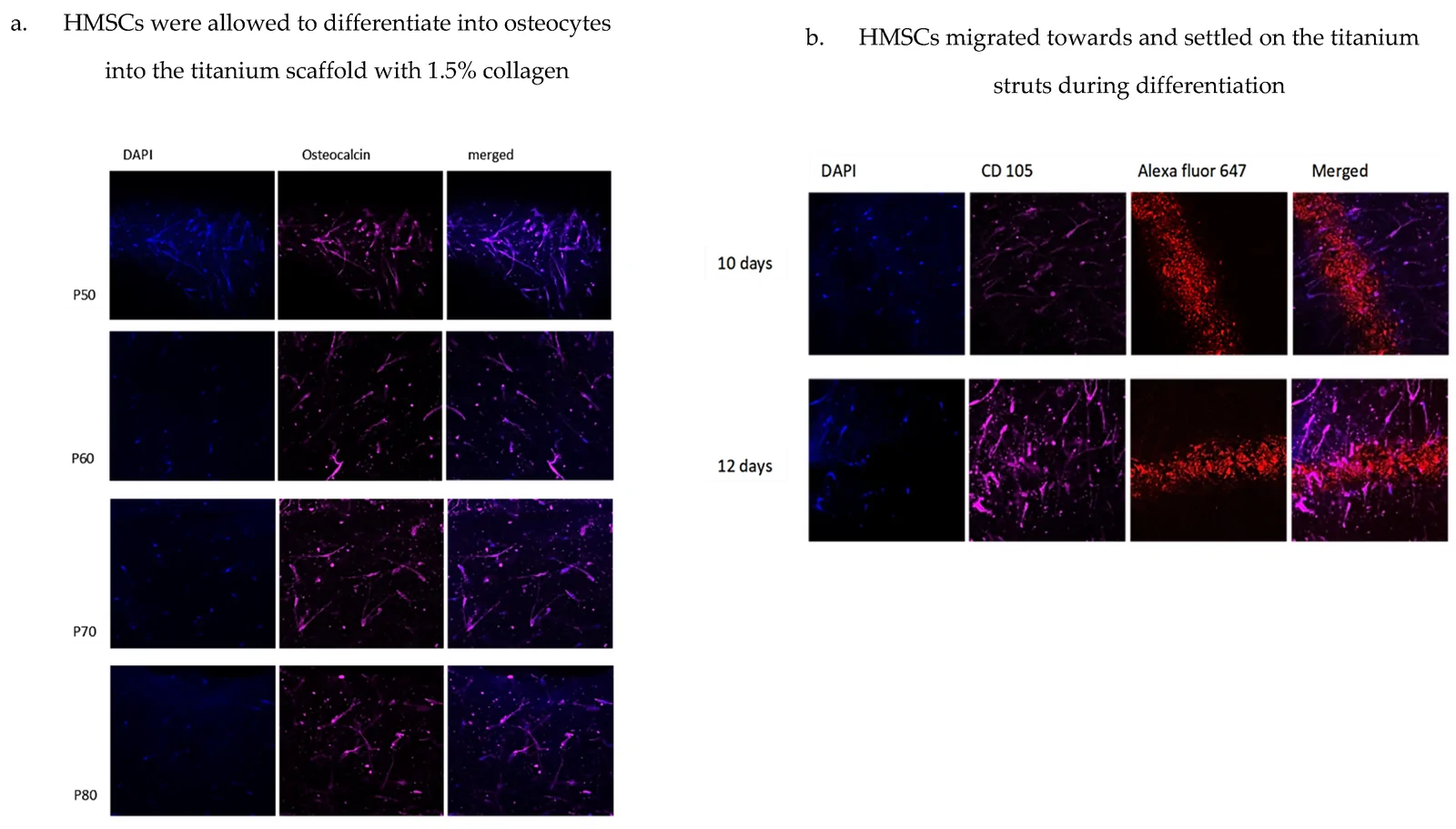
Differentiation of hMSCs towards osteoblasts within 1.5% collagen-containing titanium scaffolds. (**a**) Representative immunofluorescence images of terminally differentiated osteoblasts after 26 days of culture in the different scaffold architectures. Comparable numbers of osteoblasts were observed within the collagen matrix between the titanium struts across all scaffold types. Cell nuclei were stained with DAPI (blue), while Osteocalcin expression is shown in magenta. (**b**) Representative immunofluorescence images of early differentiating hMSCs (days 10 and 12) demonstrating active migration toward the titanium struts, where the cells preferentially adhered and colonized the scaffold surface. Cell nuclei were stained with DAPI (blue), CD105-positive cells are shown in magenta, and the titanium struts visualized using Alexa Fluor 647 are shown in red. Titanium struts were visualized using Alexa Fluor 647 staining. Images are representative of four independent donors (*n* = 4), each analyzed in triplicate.

In a second set of experiments, we introduced hMSCs into the collagen of the scaffolds and we allowed them to differentiate towards bone cells. They were stained for immunofluorescence, and pictures were obtained at different time points of 10, 12 and 30 days. Initially we took pictures of terminal differentiated cells at the collagen region rather than the titanium strain region and what was observed was that all the scaffolds presented similar osteocyte concentration which was rather strange since in our set of experiments without collagen it seems that the denser scaffold (p50) had the potentiality to retain significantly higher number of viable cells compared to the rest of the scaffolds (Figure 5a). What was done next was to stain the titanium strains with Alexa Fluor 647, which is known to interact with titanium dioxide (TiO_2_) surfaces, while HMSCs were stained with CD105 PerCP-Cy™5.5 at 10 and 12 days. What was observed in this case was the fact that stem cells were moving towards the titanium strains and they tended to coat them (Figure 5b). According to our experiments it seems that the optimum scaffold is the one with the largest titanium area, the p50.

## 4. Discussion

Advances in additive manufacturing have enabled the fabrication of anatomically precise three-dimensional (3D)-printed titanium scaffolds with precisely controlled pore size, geometry, and overall porosity [42]. This technological progress allows for the customization of implant and scaffold architecture to achieve an optimal balance between mechanical stability and biological performance. Porosity directly influences cell adhesion, migration, nutrient diffusion, and vascular infiltration [43]. Interconnected pore networks facilitate tissue ingrowth and enhance integration with host bone [40]. At the same time, excessive porosity may compromise mechanical strength, whereas insufficient porosity may hinder cellular colonization and vascularization [43].

While optimal porosity ranges for osseous regeneration have been extensively investigated in conventional lattice and porous titanium systems, considerably less attention has been given to how biologically available titanium surface area within irregular Voronoi-based LPBF architectures influences the earliest stages of stem-cell retention, survival and cell–material interaction independently of long-term mineralization outcomes [44,45]. Most previous studies have primarily focused on bulk porosity percentages, compressive strength, permeability or late osteogenic performance, whereas the immediate cellular response to differences in available titanium interface within architecturally comparable scaffolds remains incompletely characterized [19,46].

The aim of the present study was to determine the optimum titanium scaffold properties with regard to porosity and overall titanium surface of additively manufactured Ti-6Al-4V scaffolds under identical experimental conditions. Importantly, the present work did not seek to redefine universally optimal pore sizes for bone formation, nor to isolate the independent contribution of each parameter, but rather to comparatively evaluate how graded Voronoi scaffold architectures fabricated under controlled LPBF conditions influence early hMSC retention and osteogenic microenvironment formation. The optimization of scaffolds was examined in respect to their ability to retain HMSC and osteoblasts, the viability and functional stability of early human mesenchymal stem cell (hMSC) and osteogenic cell and their ability to allow the differentiation of hMSC towards bone.

An important observation of the present study is that although the P50 scaffold consistently retained significantly higher numbers of hMSCs and differentiated osteoblasts than the more porous architectures, all scaffold configurations supported osteogenic differentiation, as demonstrated by comparable osteocalcin expression. These findings suggest that scaffold architecture primarily influences early cellular attachment and retention rather than the intrinsic differentiation potential of the surviving cells. From a translational perspective, maximizing early cell retention may increase the number of viable regenerative cells available immediately after implantation, thereby creating a more favorable biological microenvironment for subsequent bone repair while preserving normal osteogenic commitment. This distinction is clinically relevant because successful bone regeneration depends not only on the differentiation capacity of individual cells but also on maintaining a sufficiently large population of viable osteogenic cells within the implanted scaffold.

A particular strength of this study is that all scaffold variants were generated from related Voronoi design principles and manufactured using the same LPBF platform and process window, thereby minimizing manufacturing-related variability and enabling biologically meaningful comparison between architectures primarily differing in designed pore volume and available titanium surface area. In addition, dimensional conformity between CAD and printed constructs was verified through optical laser scanning and nominal-to-actual deviation analysis, linking the biological findings to experimentally validated geometrical features rather than theoretical CAD designs alone.

According to our findings retention of hMSC and osteoblasts is proportionally related to the overall surface area of the scaffold. Scaffolds with a large overall surface (P50, highest titanium area/lowest porosity) consistently retained a significantly higher number of viable hMSCs and differentiated osteoblasts over 120 h incubation. Furthermore, the comparable relative growth rates observed between 24 and 120 h suggest that the superior performance of the P50 scaffold was not attributable to enhanced cell proliferation, but rather to its greater ability to retain viable cells during the initial attachment phase. This finding further supports the importance of scaffold architecture in establishing a favorable regenerative microenvironment.

Notably, among all evaluated architectures, the P50 topology consistently demonstrated superior early cellular retention and colonization behavior. To our knowledge, this is among the first studies to identify a low-porosity/high-surface-area Voronoi Ti-6Al-4V LPBF architecture as particularly favorable for early hMSC stabilization under short-term in vitro conditions. This observation is important because many contemporary scaffolds design strategies emphasize maximization of porosity for vascular penetration and permeability, potentially overlooking the critical requirement for sufficient solid interface during the initial post-implantation cellular attachment phase.

In all cases where titanium scaffolds were used there were not any significant differences regarding apoptosis or any cell-cycle distribution alterations in either the hMSC or osteoblasts, compared to the same cell population grown in cell culture dishes. In another set of experiments aiming to resemble the physical process of bone healing in the case of a bone fracture gap, 1.5% collagen was added to the scaffolds. In this case it was observed that while cell numbers within the collagen matrix were comparable across scaffold architectures, hMSCs actively migrated toward and coated the titanium struts, again favoring the P50 topology. Collectively, these results indicate that scaffold architectures with greater titanium surface area provide a more supportive early microenvironment for hMSC adhesion and short-term survival prior to osteogenic commitment.

The enhanced cell retention observed on P50 most likely results from a combination of greater surface area for attachment, more supportive local microtopography, and distinct mechanical and biophysical signals, such as differences in local stiffness and strain distribution relative to scaffolds with bigger pore diameter and less titanium struts. In addition, the intrinsic micro-roughness generated during the LPBF process may have further contributed to the enhanced early retention of hMSCs. Unlike mechanically polished surfaces, LPBF-fabricated titanium struts retain microscale topographical features that facilitate protein adsorption, focal adhesion formation, and integrin-mediated cell attachment. Consequently, the combination of the preserved laser-generated surface topography with the greater biologically available titanium surface area of the P50 scaffold likely provided a more favorable microenvironment for initial cell anchorage and early cell stabilization.

An increased strut surface provides more sites for focal adhesions and integrin-driven binding, promoting improved cell spreading and viability. The absence of scaffold-related apoptosis or alterations in cell-cycle patterns as expected, indicates that titanium is an inert material in respect to cells and the tested structural designs are cytocompatible and do not trigger early proliferative stress in hMSCs or osteoblasts. Additionally, the movement of cells from the collagen matrix onto the titanium struts suggests that cells actively migrate toward and preferentially occupy rigid surfaces (struts), even when a softer substrate is present, highlighting the critical role of surface area and surface properties in governing early cell–scaffold interactions.

Collectively, the present data suggest that early biological performance cannot be interpreted solely through nominal porosity values. Instead, the balance between pore openness and biologically accessible titanium interface appears to represent a critical design parameter in LPBF-manufactured scaffolds. The present data therefore provide experimentally supported evidence that scaffold topology and effective titanium exposure may be equally important as pore size itself during the initial establishment of a regenerative niche.

These findings provide evidence for selecting scaffold porosity based on biological considerations in load-bearing reconstructive implants. Although increased porosity is commonly encouraged to enhance blood vessel formation and limit stress shielding, our findings indicate that more compact structures may more effectively preserve and sustain implanted progenitor cells during the early phases, an aspect that could be essential for forming a functional regenerative environment, while highly porous configurations may better support subsequent tissue ingrowth. Consequently, the results support a more dynamic interpretation of scaffold optimization, where the “optimal” architecture may depend on the specific biological stage being targeted. Lower-porosity/high-surface-area designs such as P50 may be advantageous during the early cellular stabilization phase, whereas higher-porosity constructs may become increasingly beneficial during later vascularization and tissue remodeling stages.

The results of the present study should be interpreted within the context of experimental design. The biological performance of the investigated scaffold architectures was evaluated exclusively under in vitro conditions and focused on the early phase of cell–scaffold interactions. Accordingly, the present findings should be interpreted with caution when considering long-term osteogenesis, vascularization, or clinical bone regeneration. In addition, the investigated scaffold series was intentionally designed so that porosity and available titanium surface area varied simultaneously across the tested architectures. Therefore, the present study was not designed to distinguish the independent contribution of each parameter to the observed biological responses. Furthermore, scanning electron microscopy (SEM) was not performed in the present study. Future investigations combining SEM with quantitative image analysis will provide additional information regarding cell morphology, extracellular matrix deposition and cell–material interactions across the different scaffold architectures.

Taken together, the present findings provide a rationale for the development of regionally graded scaffold designs. In clinical settings, this supports the concept of regionally graded designs (greater titanium density in areas designated for cell attachment and retention, and greater porosity in zones where rapid vascular penetration is needed), thereby harmonizing early cellular stability with long-term tissue remodeling and mechanical performance. Future studies employing relevant critical-sized bone defect models are required to determine whether the enhanced early cell retention observed in the present study translates into improved bone regeneration in vivo. Future investigations should also evaluate vascularization, mechanical integration, and the effects of surface modifications, including micro- and nano-scale roughening, bioactive coatings, and chemical functionalization to distinguish the contribution of surface chemistry from that of scaffold architecture. Furthermore, longitudinal in vivo studies extending through the mineralization and remodeling phases will be essential to evaluate mature bone formation using quantitative assessments of osteogenic marker expression, extracellular matrix deposition, and the influence of physiological mechanical loading on scaffold integration.

## 5. Conclusions

Within the scaffold architecture investigated under the present experimental conditions, the titanium scaffold with the greatest available titanium surface area (P50) provided the most favorable early microenvironment for hMSC and osteoblast retention without inducing apoptosis or altering cell proliferation. These findings should not be interpreted as identifying a universally optimal scaffold architecture for bone regeneration but rather as demonstrating the biological performance of the specific scaffold designs evaluated in this study. Rather than identifying a universally optimal porosity for bone regeneration, the present study demonstrates that, within LPBF-manufactured Voronoi Ti-6Al-4V architectures, the combination of lower porosity and greater biologically available titanium surface area was associated with improved early stem-cell retention and scaffold colonization behavior. These findings support the concept that scaffold topology, specifically available solid surface for cell attachment, critically influences early stem cell behavior and should be considered alongside mechanical design criteria. The study additionally establishes an experimentally validated connection between CAD-designed architecture, LPBF manufacturing fidelity, and biological response, providing a framework for the rational design of next-generation functionally graded titanium scaffolds for bone tissue engineering.

## Figures and Tables

**Table 1:** Structural characteristics of the scaffolds.

Scaffold	Designed Porosity (%)	Mean Pore Size (µm)	Titanium Volume (mm^3^)	Available Surface Area (mm^2^)
P50	50	0.409	167.728	953.422
P60	60	0.510	128.377	796.857
P70	70	0.630	95.272	629.269
P80	80	0.770	71.162	494.966
P90	90	1.050	37.352	276.672

**Table 2:** Semi-quantitative densitometric analysis of pAkt and tAkt protein expression following normalization to GAPDH and subsequent expression relative to the hMSC control group. Values are presented as fold change (mean ± SD). Western blot band intensities were quantified by densitometric analysis. For each sample, the background-subtracted integrated density of the pAkt or tAkt band was first normalized to the corresponding GAPDH band from the same lane to control for differences in protein loading. The resulting pAkt/GAPDH and tAkt/GAPDH ratios were subsequently expressed relative to the hMSC control group, which was assigned a value of 1.00. Thus, the values presented represent fold changes relative to hMSCs after normalization to GAPDH and should not be interpreted as raw densitometric intensities.

Group	pAkt/GAPDH (Fold Change vs. hMSC)	tAkt/GAPDH (Fold Change vs. hMSC)
hMSC	1.00 ± 0.10	1.00 ± 0.08
Matrix maturation	1.85 ± 0.18	0.98 ± 0.09
Mineralization	3.10 ± 0.25	1.02 ± 0.10

**Table 3:** Relative growth rates of hMSCs cultured on the different titanium scaffold architectures between 24 and 120 h. Comparable growth rates indicate that differences in scaffold performance were primarily associated with early cell retention rather than proliferative capacity.

Scaffold	Viable Cells (24 h)	Viable Cells (120 h)	Relative Growth Rate (%)
P50	210,000 ± 18,000	255,000 ± 15,000	21.4
P60	165,000 ± 8000	180,000 ± 20,000	9.1
P70	120,000 ± 5000	130,000 ± 5000	8.3
P80	70,000 ± 3000	85,000 ± 4000	21.4
P90	30,000 ± 2000	30,000 ± 2000	0.0

## Data Availability

All data generated or analyzed during this study are included in the published article and in the Supplementary Materials.

## Supplementary Materials

The following supporting information can be downloaded at: https://www.mdpi.com/article/10.3390/jfb17090441/s1, Figure S1. Original Western blot membranes with annotated lanes and cropped regions corresponding to Figure 1d.

## Abbreviations

The following abbreviations are used in this manuscript:

Abbreviation	Full term
MSC	Mesenchymal stem cell
MSCs	Mesenchymal stem cells
hMSC	Human mesenchymal stem cell
hMSCs	Human mesenchymal stem cells
ISCT	International Society for Cell & Gene Therapy
DMEM	Dulbecco’s Modified Eagle Medium
FBS	Fetal bovine serum
3D	Τhree-dimensional
PCR	Polymerase chain reaction
FITC	Fluorescein isothiocyanate
APC	Allophycocyanin
PE	Phycoerythrin
PerCP-Cy5.5	Peridinin-chlorophyll-protein cyanine 5.5
HLA-DR	Human leukocyte antigen—DR isotype
PBS	Phosphate-buffered saline
PI	Propidium iodide
UV	Ultraviolet
DAPI	4′,6-diamidino-2-phenylindole
CAD	Computer-aided design
STL	Stereolithography
LPBF	Laser powder bed fusion
SLM	Selective laser melting
Ti-6Al-4V	Titanium–6 aluminum–4 vanadium alloy
RIPA	Radioimmunoprecipitation assay
SDS-PAGE	Sodium dodecyl sulfate–polyacrylamide gel electrophoresis
HRP	Horseradish peroxidase
IgG	Immunoglobulin G
GAPDH	Glyceraldehyde-3-phosphate dehydrogenase
PI3K	Phosphoinositide 3-kinase
Akt	Protein kinase B
ALP	Alkaline phosphatase
VEGF	Vascular endothelial growth factor
RANKL	Receptor activator of nuclear factor kappa-B ligand
OPG	Osteoprotegerin
TPMS	Triply periodic minimal surface
3D	Three-dimensional
mTi-6Al-4V	Manufactured titanium–6 aluminum–4 vanadium alloy
TiO_2_	Titanium dioxide
