# Supplementary material for: Influence of Ti-6Al-4V Scaffold Architecture on Early Cellular Responses Relevant to Bone Regeneration
Source: J Funct Biomater. 2026 Sep 1;17(9):441. doi: 10.3390/jfb17090441 (PMC13608623; doi:10.3390/jfb17090441)
Supplement: Supplementary file 1 [file jfb-17-00441-s001.zip › jfb-4446289-supplementary.pdf]

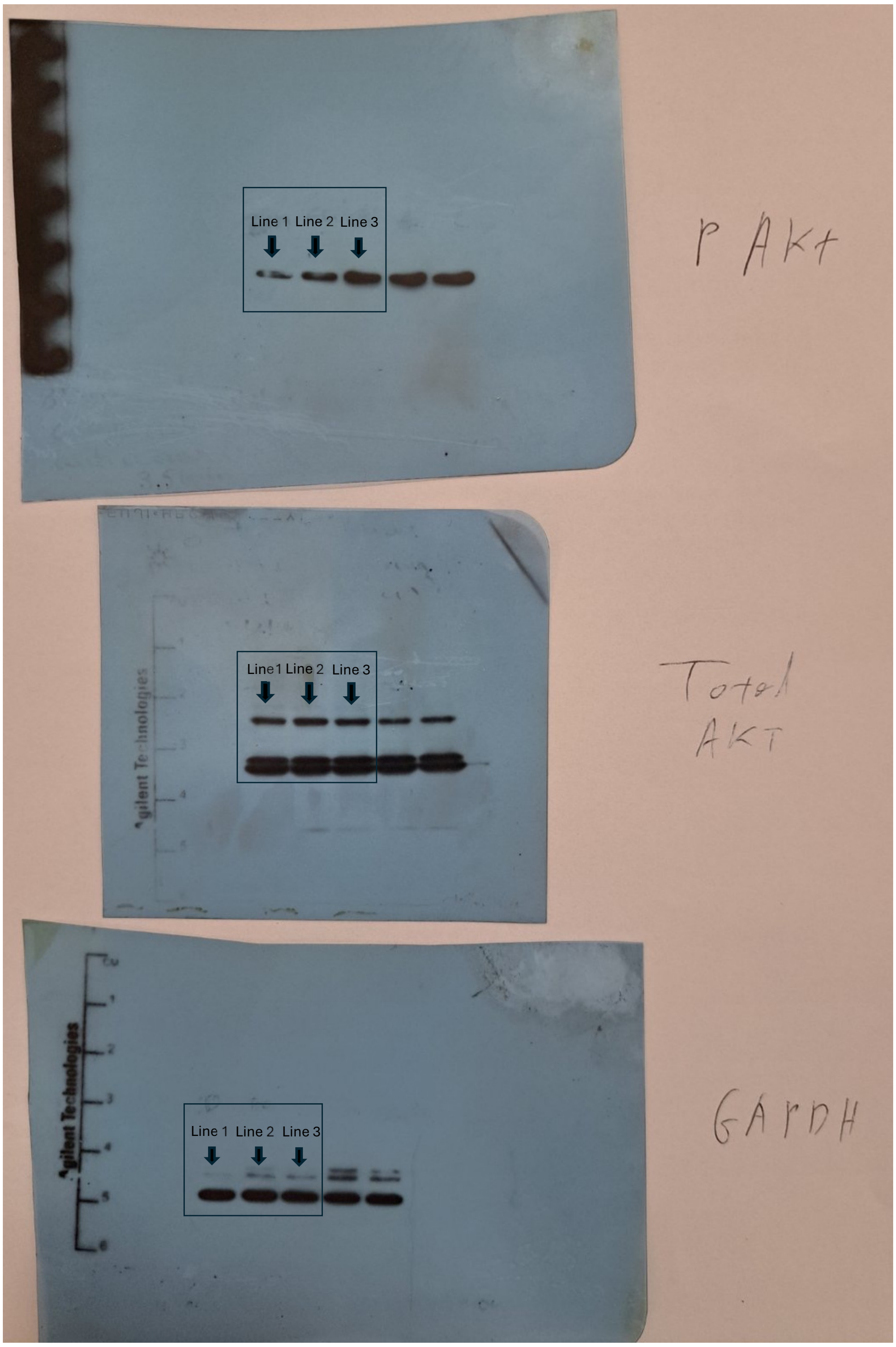

Figure 1d

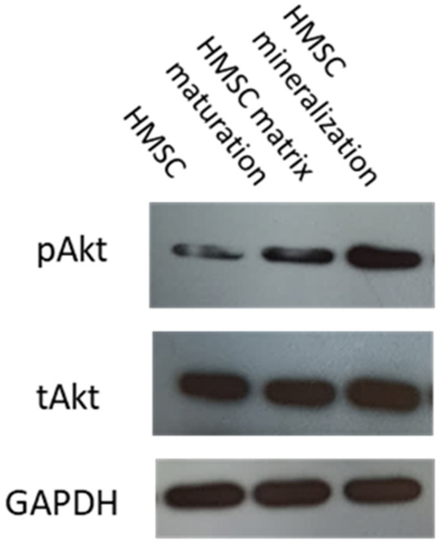

Figure S1. **Original Western blot membranes with annotated lanes and cropped regions corresponding to Figure 1d.** The boxed regions indicate the portions of the original membranes used to generate the representative cropped bands shown in Figure 1d. Lane 1 = undifferentiated hMSCs (day 0); Lane 2 = hMSCs during matrix maturation (day 10); Lane 3 = hMSCs during mineralization (day 20). Arrows indicate the corresponding lanes used for the analysis of phosphorylated Akt (pAkt), total Akt (tAkt), and GAPDH. GAPDH was used as the loading control for normalization of pAkt and tAkt band intensities.
